# Sudden Onset of Intramural Duodenal Hematoma During Hemodialysis in Hypertensive Patient

**DOI:** 10.1002/jgh3.70078

**Published:** 2024-12-16

**Authors:** Hideaki Kazumori

**Affiliations:** ^1^ Department of Gastroenterology Matsue Seikyo General Hospital Matsue Shimane Japan

**Keywords:** hemodialysis, hypertension, intramural duodenal hematoma

## Abstract

A 79‐year‐old man undergoing treatment with warfarin for atrial fibrillation and hemodialysis for renal failure was transferred to our hospital for rehabilitation. During a maintenance hemodialysis session, blood pressure was shown to be elevated and an intramural duodenal hematoma suddenly occurred. After 3 days, the hematoma had enlarged and angiographic embolization was performed, with complete resolution noted after 2 months. Occurrence of an intramural duodenal hematoma during hemodialysis is rare. However, acute abdominal pain with symptoms indicating obstruction in patients undergoing such treatment should raise suspicion regarding an intramural duodenal hematoma. Although conservative treatment is often effective for a nontraumatic intramural hematoma, early angiographic embolization is preferred when disruption of anticoagulant therapy is difficult or for patients with failed medical treatment.

## 1 Introduction

An intramural duodenal hematoma of the gastrointestinal tract is an uncommon condition. It most often occurs in children and young adults following a blunt abdominal trauma [1, 2], though rarely under a nontraumatic condition, with some studies noting occurrence in association with various types of pancreatic disease, connective tissue disease, a duodenal ulcer, and a pancreaticoduodenal aneurysm [3, 4, 5, 6, 7, 8]. Other causes of nontraumatic intramural duodenal hematoma reported include bleeding tendency, such as coagulation disorders or complications associated with anticoagulant therapy [9, 10].

Chronic renal disease and hemodialysis are risk factors for gastrointestinal hemorrhage, while diabetes mellitus, coronary artery disease, cirrhosis, and use of nonsteroidal anti‐inflammatory drugs are also each known to increase the risk of gastrointestinal hemorrhage in renal disease patients receiving hemodialysis [11]. Although several cases of nontraumatic intramural esophageal hematoma in patients undergoing hemodialysis have been reported [12], no known findings indicating an association of a nontraumatic intramural duodenal hematoma with hemodialysis have been reported.

A unique case of sudden onset of intramural duodenal hematoma during hemodialysis in a hypertensive patient is presented.

## 2 Case Report

A 79‐year‐old man was transferred to our hospital for rehabilitation following thoracic aortic aneurysm treatment with a synthetic graft 1 month prior. There was a history of hypertension and diabetes treatment, though he had been receiving oral medication and blood pressure was good. Two days after the operation, a cardiogenic embolism was noted, despite warfarin treatment for atrial fibrillation. Postoperative renal failure was also present and maintenance hemodialysis was necessary.

**FIGURE 1:**
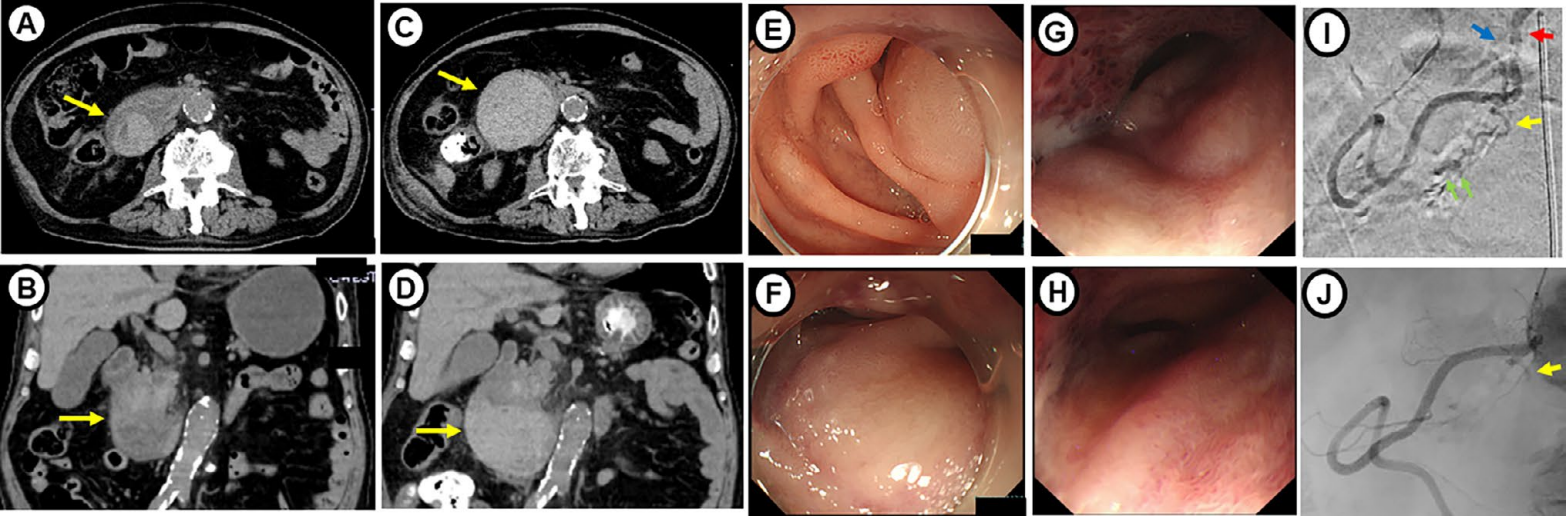
(A, B) CT findings revealed a mixed hyperdense and hypodense mass in the second portion of the duodenum (arrow). (C, D) CT findings obtained after 3 days revealed that the hematoma had become enlarged (arrow). (E, F) Esophagogastroduodenoscopy showed an intramural duodenal hematoma obstructing the second portion of the duodenum (standard endoscopic view obtained with GIF‐Q260J, diameter 9.9 mm, Olympus Corp., Tokyo, Japan). (G, H) Findings obtained deeper into the duodenum (thin endoscopic view obtained with GIF‐XP290N, diameter 5.8 mm, Olympus Corp, Tokyo, Japan). (I) Angiography findings. Red arrow, gastroduodenal artery; yellow arrow, anterior superior pancreaticoduodenal artery (ASPDA); blue arrow, posterior superior pancreaticoduodenal artery; extravasation from ASPDA branches was noted (green arrows). (J) Findings obtained following embolization of the ASPDA showed no evidence of active bleeding.

Following admission, the only notable condition was sleep deprivation caused by pain in the operation scar area, which caused the patient to become irritable with inadequate sleep. Routine hemodialysis using heparin, scheduled for 3 h, was performed, though blood pressure of 200/110 mmHg was noted at the start of treatment, as compared to the usual level of approximately 130/80 mmHg. Although nicardipine hydrochloride was administered, the level remained high. Two hours after starting hemodialysis, blood pressure became further elevated to 230/120 mmHg, along with a sudden onset of severe right epigastric pain and vomiting. Laboratory results showed hemoglobin at 9.8 g/dL and a prothrombin time‐international normalized ratio (PT‐INR) of 2.75. CT indicated a mixed hyperdense mass measuring 46 × 47 mm in the second portion of the duodenum (Figure 1A,B). Furthermore, esophagogastroduodenoscopy findings showed edematous dark red mucosa without a mucosal defect in the second portion of the duodenum, with the lumen nearly obstructed, while the endoscope was not able to pass through. Diagnosis of an intramural duodenal hematoma was confirmed based on endoscopy findings (Figure 1E–H). Warfarin effects were inhibited by use of vitamin K and PT‐INR showed recovery to 1.28 on the following day. The next hemodialysis session was planned for 2 days after occurrence and nafamostat mesylate was used as an anticoagulant to prevent additional hemorrhaging. After 3 days, anemia progressed with hemoglobin at 7.7 g/dL and the hematoma had enlarged to 80 × 70 mm (Figure 1C,D). Enhanced CT showed weak focal enhancement within the hematoma, suggesting continuous hemorrhaging from duodenal blood vessels, such as the anterior superior pancreaticoduodenal artery (ASPDA) or posterior superior pancreaticoduodenal artery (PSPDA). Angiography was performed, which showed extravasation from the branches of the ASPDA, while none was seen from the PSPDA. Therefore, embolization of the ASPDA was performed for early resumption of anticoagulation. Complete resolution of the hematoma was noted after 2 months.

## 3 Discussion

The present patient had never undergone an endoscopic procedure for the duodenum, nor been complicated with a coagulation disorder, pancreatic or connective tissue disease, and showed no history of trauma. An intramural duodenal hematoma occurred after beginning hemodialysis, with that treatment considered to be a direct trigger for its formation. To the best of our knowledge, this is the first report of occurrence of a nontraumatic intramural duodenal hematoma during hemodialysis.

Although some reports of duodenal hematoma development in patients who had received hemodialysis have been reported [4, 5, 6], onset was noted following an endoscopic hemostatic procedure for a duodenal ulcer condition with active hemorrhaging. In these cases, it has been suggested that the mechanism involved in endoscopic rupture of an arterial blood vessel caused by needle injection might also cause subsequent formation of a hematoma in predisposed individuals, such as those with chronic renal failure and undergoing hemodialysis.

The present patient was treated with warfarin, and elevation of PT‐INR to 2.75 was noted. Two hours after starting hemodialysis, sudden onset of severe right epigastric pain and vomiting occurred. Chronic renal disease and hemodialysis are known to be risk factors for gastrointestinal hemorrhage [11], and it is considered that in addition to warfarin, heparin use during this treatment possibly facilitated the hemorrhage in the present case.

On the day of occurrence, blood pressure was elevated to a level much higher than usual despite nicardipine hydrochloride administration. It was thus considered that hemorrhaging caused by arterial collapse might have been induced by sustained high blood pressure. Volume overload and increased arterial stiffness are important factors responsible for hypertension noted in chronic renal failure patients on hemodialysis [13], and those might have been factors related to refractory hypertension in the present patient. An additional notable condition was irritability due to sleep deprivation caused by pain in the prior surgical scar area. Indeed, sleep deprivation is known to be associated with relevant alterations in neuroendocrine function and may play an important role in acute blood pressure elevation [14].

In patients with intramural duodenal hematoma formation caused by acute pancreatitis, leakage of pancreatic enzymes in association with pancreatitis can cause injury to duodenal blood vessels, followed by formation of pseudoaneurysms. In contrast, a nontraumatic intramural duodenal hematoma caused by a nonpancreatic disease usually originates from small blood vessels in a submucosal layer, for which conservative medical management is often effective [10]. Nevertheless, patients with failed conservative medical management are occasionally encountered, with surgical intervention performed for those with a significant intramural hemorrhage, bowel perforation, or identifiable risk of ischemia [5, 9]. In the present case, though inhibition with anticoagulation therapy was attempted, enlargement of the hematoma continued. Angiography findings showed that the pseudoaneurysm was not different from those seen in cases caused by pancreatic diseases. Extravasation from ASPDA branches was revealed, which, along with the long‐term history of diabetes and hypertension, may have led to atherosclerosis, followed by blood vessel fragility. Because of the presence of atrial fibrillation and a history of cerebral embolization, angiographic embolization of the ASPDA was preferred and it was considered important to restart warfarin as soon as possible. Hemodialysis with use of nafamostat mesylate was performed for only one session, then the anticoagulant was switched back to heparin following embolization, with complete resolution of the hematoma noted 2 months later.

Although a nontraumatic intramural duodenal hematoma is rare, acute abdominal pain with symptoms of obstruction in patients during hemodialysis should raise suspicion regarding a nontraumatic intramural duodenal hematoma. Conservative management is generally the initial treatment of choice. However, in cases that show difficulty with disruption of anticoagulant therapy or have failed medical treatment, early angiographic embolization should be considered as an effective treatment option. Furthermore, in patients with a history of cerebral embolization, it is important to consider restarting warfarin as soon as possible.

## Consent

Written consent for publication was obtained from the patient.

## Conflicts of Interest

The author declares no conflicts of interest.
